# Development of the automated software and device for determination of wicking in textiles using open-source tools

**DOI:** 10.1371/journal.pone.0241665

**Published:** 2020-11-16

**Authors:** Predrag M. Milanovic, Snezana B. Stankovic, Milada Novakovic, Dragana Grujic, Mirjana Kostic, Jovana Z. Milanovic

**Affiliations:** 1 Faculty of Technology and Metallurgy, University of Belgrade, Belgrade, Serbia; 2 Faculty of Technology, University of Banja Luka, Banja Luka, Bosnia and Herzegovina; 3 Innovation Centre, Faculty of Technology and Metallurgy, University of Belgrade, Belgrade, Serbia; The University of Alabama, UNITED STATES

## Abstract

The development of automated software and the device for determination of wicking of textile materials, using open-source ImageJ libraries for image processing, and newly designed additional algorithm for the determination of threshold, is presented in this paper. The description of the device, design of the open-source software “Kapilarko”, as well as an explanation of the steps: image processing, threshold determination and reading of wicking height, are provided. We have also investigated the possibility of using the artificial neural networks for automatic recognition of the wicking height. The results showed that the recognition of the wet area of the sample, based on the application of artificial neural networks was in a very good agreement with the experimental data. The device's utility for the measurement of wicking ability of textile materials was proved by testing various knitted fabrics. The constructed device has the advantages of providing automated measurement and minimization of the subjective errors of the operators; extremely fast or long-term measurements; digital recording of results; consistency of experimental conditions; possibility of using water instead of colors and, last but not least, low cost of the device. Considering the importance and frequent measurements of wicking ability of textile materials, the advantages of the presented device, as well as the fact that commercial software without publishing the source-code, are used for most of the available devices, we believe that our idea to design the automated software and device by applying the "open-source" approach, will be of benefit to scientists and engineers in using or improving wicking experiments.

## Introduction

One of the most widespread applications of textile materials is certainly in the clothing manufacture. Clothing has an important role in keeping the body comfortable by removing sweat [1]. In everyday life and in an activity when the metabolism is very high, people sweat and perspiration spreads all over the skin, that’s why, clothes should transfer quickly the sweat outside to provide physiological comfort for the wearers, especially in sportswear, underwear, working garment or protective clothing, and finally to have a beneficial effect on human health and wellbeing [2–6]. Given that the liquid transfer through textiles is a critical factor that affects tactile and sensorial comfort, it is important to understand liquid transport mechanisms [7–9].

Generally, in order to characterize the liquid water uptake, a measurement of wicking (capillary rise), which includes observing and measuring the capillary flow of a liquid, which happens because of capillary forces, when the yarn/fabric is placed perpendicularly to a liquid bath, has been applied [8, 10, 11]. The capillary force depends on the radius of the capillary channel and the contact angle between liquid and capillary channel as well as on rheological properties of the liquid [12].

In order to study experimentally the wicking ability of textiles, different techniques have been employed. The first techniques were based on using colored liquids [13, 14] and these techniques have been used by many researchers [10, 15–17]. However, the application of dyes has a lot of disadvantages because the kinetics of water can be more important than that of dye and the diffusion coefficient presents the value of the diffusion coefficient of the dye, not of the liquid [10, 13, 14]. Ferrero et al. used potassium chromate aqueous solution, instead of dye solution, because it is an inorganic salt without affinity toward synthetic fibers [11].

Many researchers [18–20] used a balance in their studies to measure the impregnation liquid mass variation in the solid structure. Methods with a balance usage are unable to determine the equilibrium height and the quantity of liquid absorbed by the textile at different heights. There are some experimental installations that are made by using balance and camera [21–23]. There are a lot of variations: the balance is above the sample and the total mass of wet sample is measured [21], and the fluorescence [22], dye [23] or UV light [23] is used to emphasize the difference between dry and wet area. Achour et al. investigated the kinetics of the water capillary rise into the cotton knitted fabric by using an ameliorated experimental system with the UV lighting system in a darkroom, but without the addition of dye [24]. Cause et al. developed a capillary rise experimental installation, also placed inside the darkroom, to record simultaneously the flow front position, by using an inert fluorescent dye, and the uptake fluid mass [23].

Other techniques consist of measuring water transport along textile fibers by an electrical capacitance and it is based on the electrical resistance principle [25–27]. This technique consists of the construction of an apparatus with a specially designed electrical amplifier circuit and condenser electrodes, between which sample fibers are set. Although this method is also unable to measure the liquid height at various levels, it permits only a global view of the evolution of liquid transport [28, 29].

One of the first applications of an optical system linked with an image analysis, based on the analysis of CCD images taken during the capillary rise of a colored liquid in polyester yarn was done by Perwuelz et al. [10, 16]. The results obtained by the image analysis technique depend on the resolution, the quality of images and the light source. However, the main drawback of this method is the fact that the addition of the dye changes the liquid properties and modifies its velocity [10]. Some researchers use the relatively expensive CCD digital cameras [23, 30]. In addition, most of the work is done by using a commercial software (Matlab [21, 23], LabVIEW [22], etc.), and without publishing source code.

Taking into consideration great importance and frequent need for determination of the wicking ability of textile materials, as well as all disadvantages of the above mentioned techniques, our idea was to design the automated software and device for determination of wicking in textiles by "open-source" approach. By using these open-source scientific tools available to everyone, researchers and engineers can construct and adapt the wicking device according to their own needs. According to the Open Source Definition [31], free and open-source computer software is available in source code form and can be used, studied, copied, modified, and redistributed without restriction, or with restrictions that only ensure that further recipients have the same rights under which it has been obtained [32]. A great number of various open-source do-it-yourself devices, such as a potentiostat [33–35], syringe pump [36], a colorimeter [37], device for measuring thermal resistance of textiles [38], additional software improvements for microscopes [39], and the pH-stat [32] have been developed, but to the best of our knowledge, the idea to construct an open-source device for determination of wicking ability has not still presented.

Artificial neural networks (ANN) have been widely used to model some of the human activities in many areas of science and engineering. Today, neural networks are used for solving many data analysis such as sales forecasting, customer research, data validation, and risk management. ANN also found their application in computer vision, natural language understanding, and many other every-day areas [40]. One of the distinctive feature of the ANN is its ability to learn from experience and examples and then to adapt according to changing situations [41].

In this work, the automated software and device for determination of wicking in textiles developed in our laboratory by using open-source ImageJ libraries for image processing, and newly designed additional algorithm for the determination of threshold were presented. In addition to a description of the device and design of the open-source software called “Kapilarko”, the explanation of steps—image processing, determining the threshold, reading wicking height and artificial neural networks application were provided in this work. The device's utility was demonstrated on a range of different knitted fabrics. We have also investigated the possibility of using available tools from the field of the neural networks for the threshold determination for recognizing wet and dry areas of samples, with the aim of automatic recognition of wicking height.

## Materials and methods

### Design

**Fig 1:**
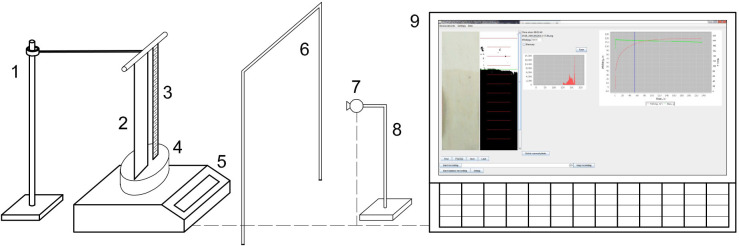
Scheme of the system for the determination of wicking, which includes 1. **Sample holder, 2. Sample; 3. Ruler; 4. Dish with water, 5. Balance; 6. Lighting; 7. Camera; 8. Camera holder and 9.** PC, with screen shot recorded during the measurement of wicking.

The design aim was to create a device for the determination of wicking of textiles. We made a decision that wicking should be recorded by a personal computer using a webcam and the software which should be able to work independently. The structure of the system for the determination of wicking, is shown in the Fig 1, and a description of each component is given below.

The sample (2) was mounted on the frame and hanged on the pillar/sample holder (1) above the dish with water (4), placed on the balance (5). The sample was positioned according to the modified Standards: AATCC Test Method 197, Vertical Wicking of Textiles and ISO 9073–6:2000, so it was immersed 2 cm in the liquid. In our experiments distilled water was used as wicking liquid, although other liquids could be also used.

Parallel to the sample, the ruler (3) was placed which enabled the calibration of lengths from the pictures to obtain the wicking height (mm).

The very important aspect of this set is that the lighting should be constant during the experiment. In order to provide the constant lighting, we have used two led strips (6) (neutral color 4000 K, 40 cm) vertically mounted between the camera (7) and sample (2) in the way not to obscure the sample. Fine adjustment of the distance between the sample, the lighting and camera can be done depending on the light (intensity and color) and the sample (thickness, color) which is to be tested. In order to present the system for the determination of wicking as clearly as possible, the layout of the components (sample, lighting, camera) applied in our experiments was given in S1 Fig.

The led strips have to be of such intensity to provide the constant lighting so as to overcome changing of the ambient light during the experiment. In addition, the lighting has to be constant along the sample. If these conditions are not met, it may lead to the incorrect recognition of wet/dry areas due to the shadows or different intensity of the wetted areas of the sample during the measurement.

**Fig 2:**
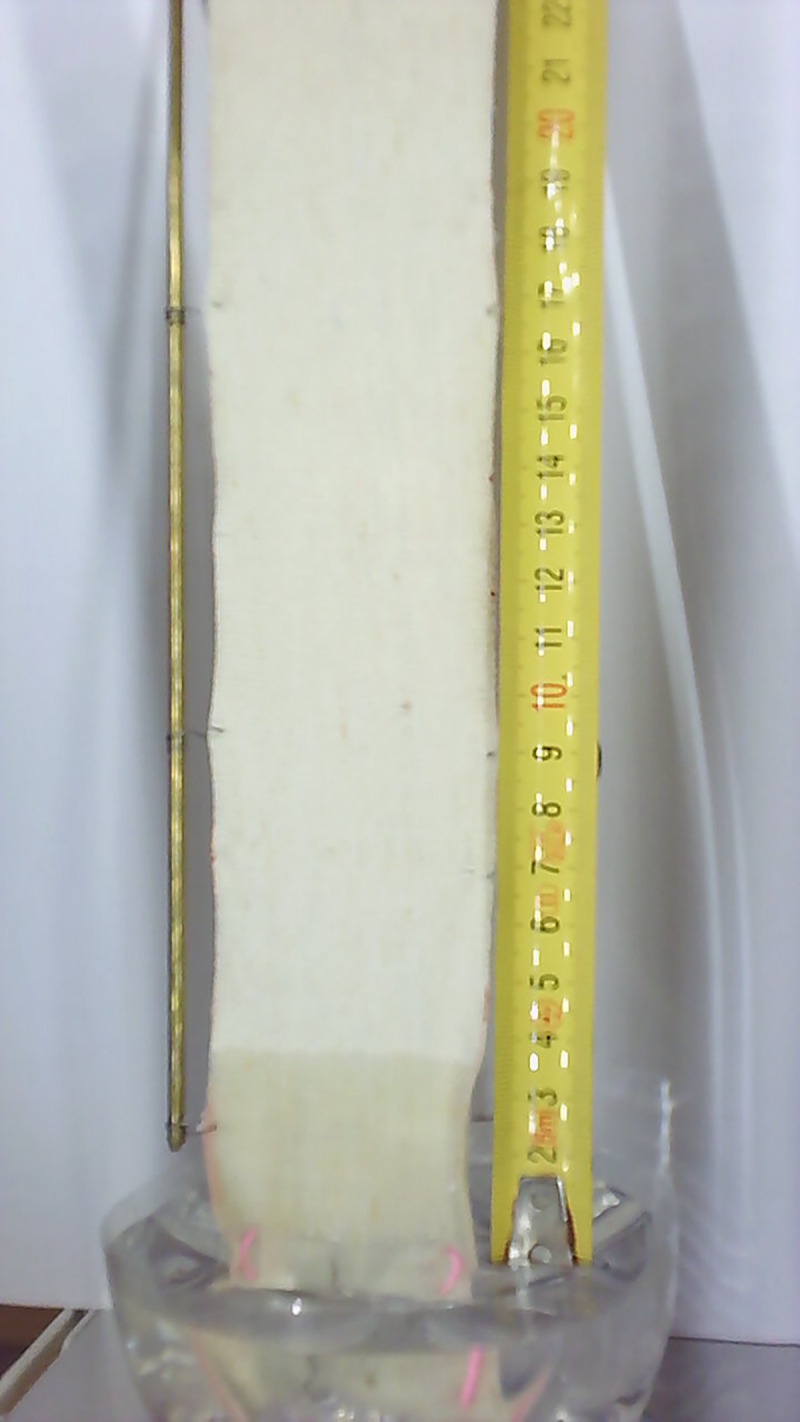
Example of the recorded image during determination of wicking of the hemp knitted fabric.

Images recorded by the camera (7) placed on the camera holder (8), behind the lighting (6), are processed in real-time and results are presented on PC (9), in the open-source software “Kapilarko”. The software is able to acquisition pictures from a webcam (7) and mass from a balance (5), as well the data from Arduino microcontrollers [42] with appropriate sensors, as will be described later. The basic assumption is that the camera (7) and sample (2) are still and with no movement of any part of the system Fig 2. Before image processing, the height and width of the section of the image processed should be calibrated and defined.

Our experiments were performed in the transparent box (120 cm x 60 cm x60 cm) to prevent intensive airflow. During the experiments, while the images were recording, the measurements from the balance and humidity and temperature of the air were simultaneously recorded, in 2 minute intervals for additional mathematical processing.

#### Open-source “Kapilarko” software

The main part of the whole system is the open-source software called “Kapilarko”. This software is written in Java. It synchronize the work of camera, data acquisition from the balance and the temperature and humidity sensors with mathematical calculations in image processing presenting the results in the graphical user interface (GUI). For creating GUI the SWING—a platform-independent Graphical User Interface for Java programs is used.

To show the current capabilities of the software, the several screenshots are presented in S1 File.

The communication between the PC and sensors was done by proxy (Arduino Nano microprocessor board) which is established through a platform-independent serial port access library for Java jSerialComm [43] (GNU General Public License v3.0).

For creating graphs, an open-source framework for the programming language Java [44] was used. It was distributed under the terms of the GNU Lesser General Public License (LGPL).

Obtained data and calculated values of the wicking height are available for use in external applications (mathematical and statistical software) through export capabilities of Kapilarko.

During work on “Kapilarko”, only platform-independent frameworks and modules available under some of open-source license were used. Therefore, the whole system works on all operating systems which support Java with the exception of a balance since only Windows drivers for the balance RADWAG AS 220.R2 existed, so the acquisition of the mass data was exclusively possible on Windows operating system. All other parts of the system were successfully tested on Debian 10 system [45] and Ubuntu 18.04 [46].

### Video and image acquisition

For communication with web camera Webcam Capture API library was used. It was published under the MIT License (MIT).

Images were regularly recorded in resolution 720 x 1280 pixels in separate PNG files. These files enable recalculations with revised starting parameters at any time.

#### Wicking recognition

The low-cost CANYON camera (CNE-CWC3, 2 Mpixel) was used. It was fixed to the construction holding a textile sample (with the end immersed in water). For wicking recognition, the special purpose code was developed. The process of wicking recognitions was divided into few steps: defining a section of images which would be of interest; analyzing the histograms of recorded pictures and calculating the threshold; determination which pixels represent wet or dry part of the sample and determination of wicking height.

### Artificial neural network—Neuroph

One of the simplest class of feedforward ANN–a multilayer perceptron with backpropagation (MLP) is used in this work. For its implementation Neuroph lightweight framework was applied. Neuroph is a software consisted from lightweight Java neural network framework and GUI tool that supports creating, training and saving neural networks. Neuroph Java neural network framework for developing common neural network architectures implements basic neural network concepts like an artificial neuron, neuron layer, neuron connections, weight, transfer function, input function, learning rule, etc. Neuroph supports common neural network architectures such as Multilayer perceptron with Backpropagation, Kohonen, Hopfield networks and many others [47].

It is an open-source project licensed under the Apache 2.0 License. It has developed GUI, but in this work, we used only libraries that provide the core functionality of Neuroph framework.

### Control of lighting

Simple, but very important component of the system is the control of lighting. Two 40 cm long led strips normal color (4000 K), powered with 12 V and mounted on two vertical supporting pillars were used as the lighting. The intensity and direction of light can be changed by an adequate position of the movable supporting pillars. If it is necessary (in case of thin sample) an appropriate background, located a few centimeters behind the sample, can be used.

### Humidity and temperature acquisition

Module for reading the humidity and temperature of the working space is connected to the computer by USB cable. This module has a simple GUI interface (independent of the “Kapilarko” software) which automatically reads and records the temperature and humidity of the air in 2 minute intervals.

DHT22 sensor was used for determining the temperature and humidity. The DHT22 is a basic, low-cost digital temperature and humidity sensor. It uses a capacitive humidity sensor and a thermistor to measure the surrounding air and spits out a digital signal on the data pin (no analog input pins needed).

To accomplish communication with DHT22 sensor an ATmega328-based Arduino Nano (arduino) was used. The code is also available on “Kapilarko” repository https://gitlab.com/mpele/Kapilarko.

### Balance

In order to obtain very useful data about the change in mass during the experiment, we added the ability to record data from a balance. High precision balance RADWAG AS 220.R2 connected with the USB cable to the computer was used in this experiment. Since this balance has only drivers for connecting on Windows operating system, our system couldn’t work at full capacity under Linux.

The “Kapilarko” software automatically reads the mass from the balance, records it in the file and presents the values on the graph.

### Sample preparation

The sample preparation was conducted to the modified standards AATCC Test Method 197, Vertical Wicking of Textiles and ISO 9073–6:2000. The program have to be started just before the sample has been immersed in water, and the camera records the sample with any changes occurring during the water uptake. Intervals for taking and storing photos can be adjusted to a minimum of 1 s. If necessary, the interval for taking pictures can be increased during the measurement.

In this paper, wicking ability was determined on different fiber content knitted fabrics: Cotton, Bamboo, Hemp, and blended composition Cotton/Bamboo, Cotton/Polyester and Hemp/Acrylic. Three measurements were done for each fabric. The measured results can be presented in the graph of the time-dependent progression of water which gives detailed information on the wicking of the fabric. It is also very suitable for comparing the behavior of textile materials of different chemical composition and structure [21–23].

## Results and discussion

“Kapilarko” source code is available on the link.

### “Kapilarko” software–Controller of the system

In order to synchronize all the components and to enable easy work, the custom software was made. This provided a user-friendly graphical interface for everyday work on the determination of wicking of textiles. The software Kapilarko was published under open-source MIT License, and therefore everyone could use it and/or adapt it for their needs.

**Fig 3:**
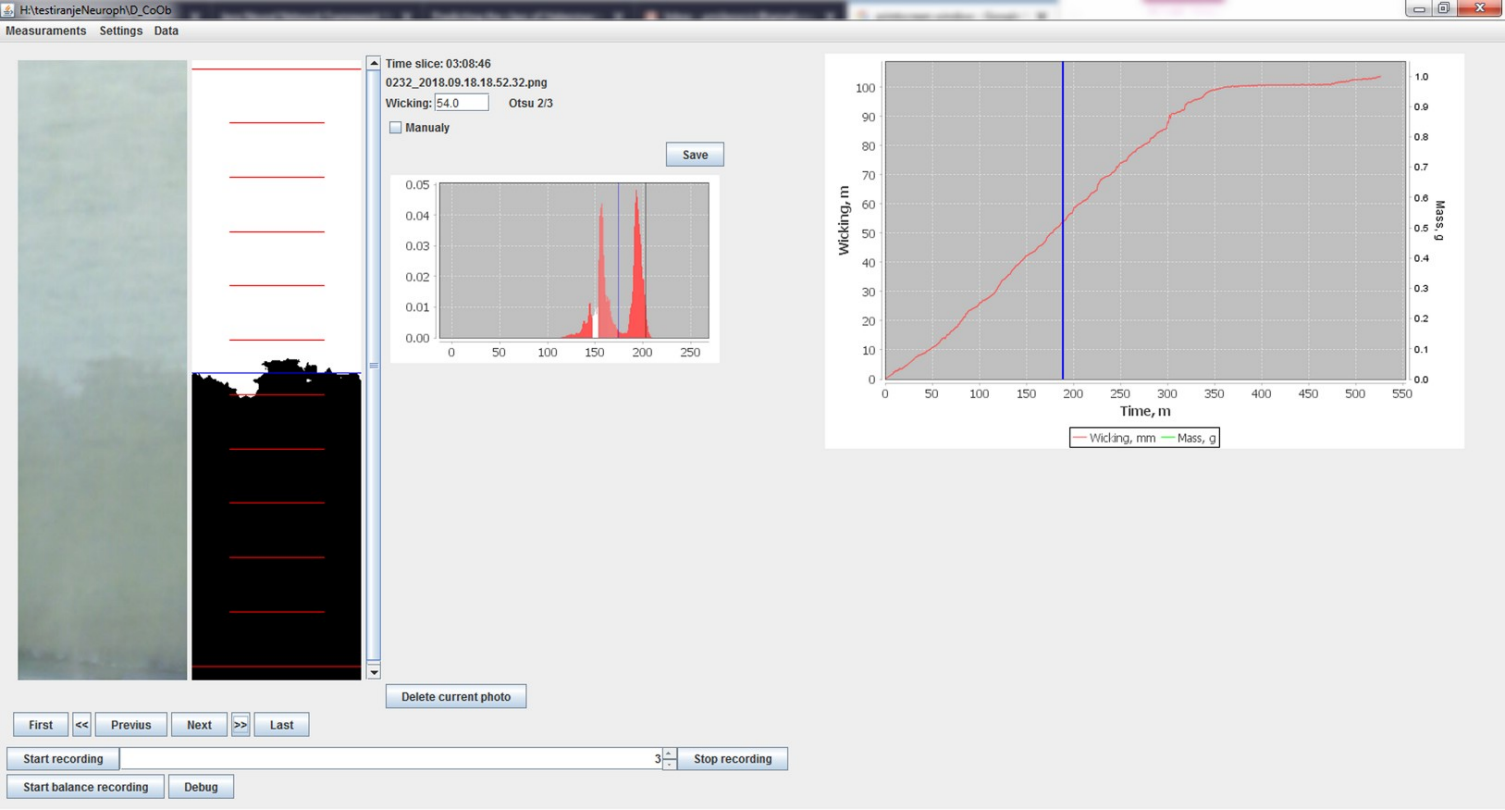
Screenshot recorded during determination of wicking of cotton knitted fabric.

The frequency of image recording can be mainly controlled by “Kapilarko”. Since the images were stored on the hard drive of the computer, they could be used later for additional analyzing and further processing. After the image was recorded, the defined section was analyzed the histogram was created, the threshold value could be determines and wicking height calculated (Fig 3). Since all calculated values are presented on the graph the operator can easily visually determine in which phase the current experiment is and accordingly, to define the frequency of recording.

Graphical User Interface on the main screen provides the additional data about the currently selected image—the part of the recorded image, and histogram with the marker for the threshold used (Fig 3). Parallel with the image, the processed image section with dark pixels for the wet and the white ones for the dry portion of the sample is shown together with the mark for the determined wicking height. Collected information is available for all recorded images with a clear mark on the main graph with the values of wicking height (Fig 3). Available information helps the operator to understand the whole process.

“Kapirlarko” can also be used for starting the acquisition of data from a balance. In addition, “Kapilarko” provides additional necessary tools for work: interface for defining section which will be processed, with the ability to calibrate distances on the image, live preview which helps for setting camera and lighting, and interface for choosing an appropriate threshold, which will be described in detail below.

“Kapilarko” provides the ability to export data in a format which could be used in other specialized software and tools. Example of excel file generated by “Kapilarko” is presented in S2 File.

### Working principle of system for determination of wicking

**Fig 4:**
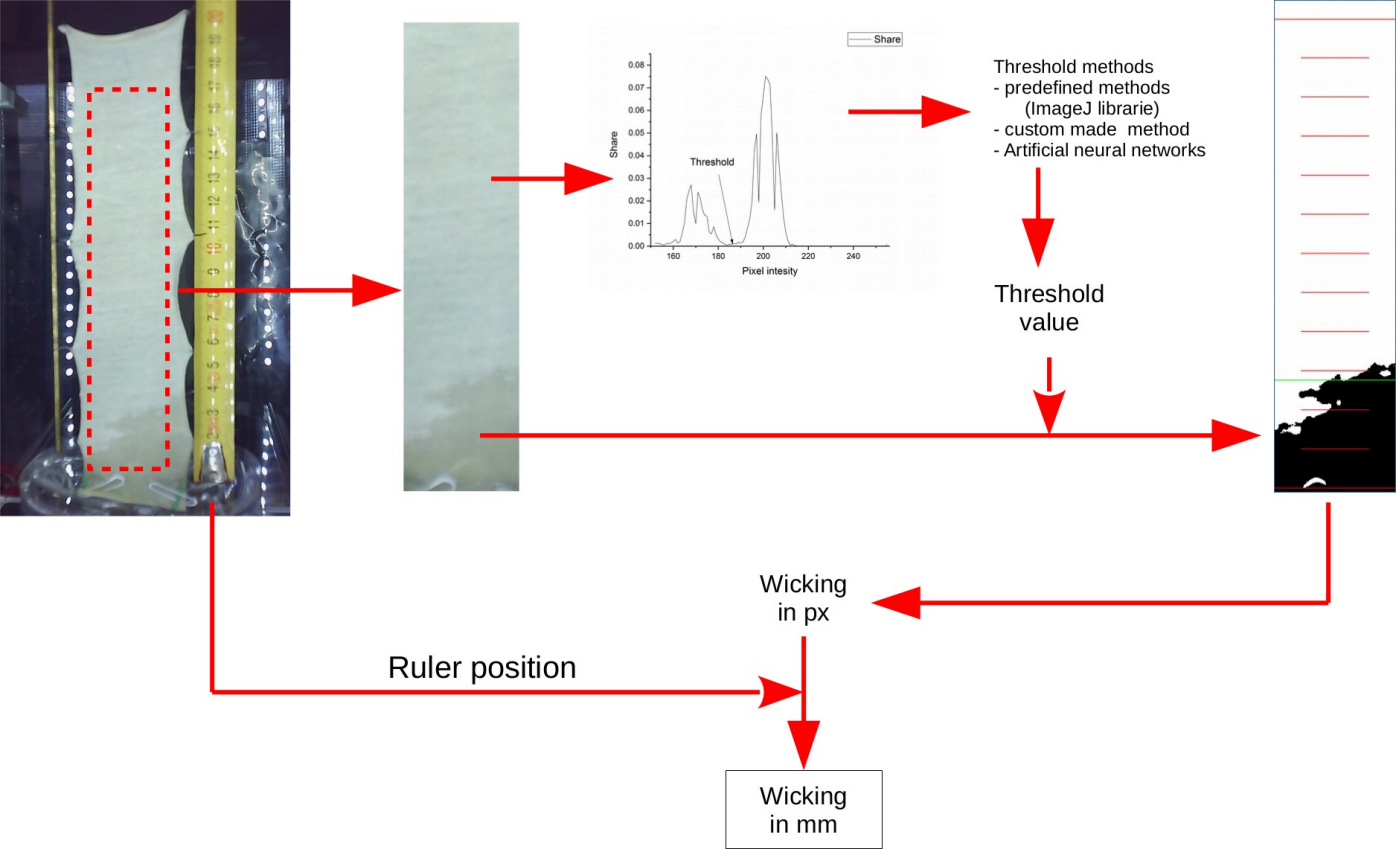
Principe of determination of wicking.

General principle of the determination of wicking is presented on the Fig 4. All the steps are described in details in this chapter.

#### Taking images

In order to record an image correctly, it is important to provide the constant lighting along the sample, as well as during the experiment. Usage of led strips (Chapter Control of lighting) gives the possibility to avoid work in a dark room [23, 24].

**Fig 5:**
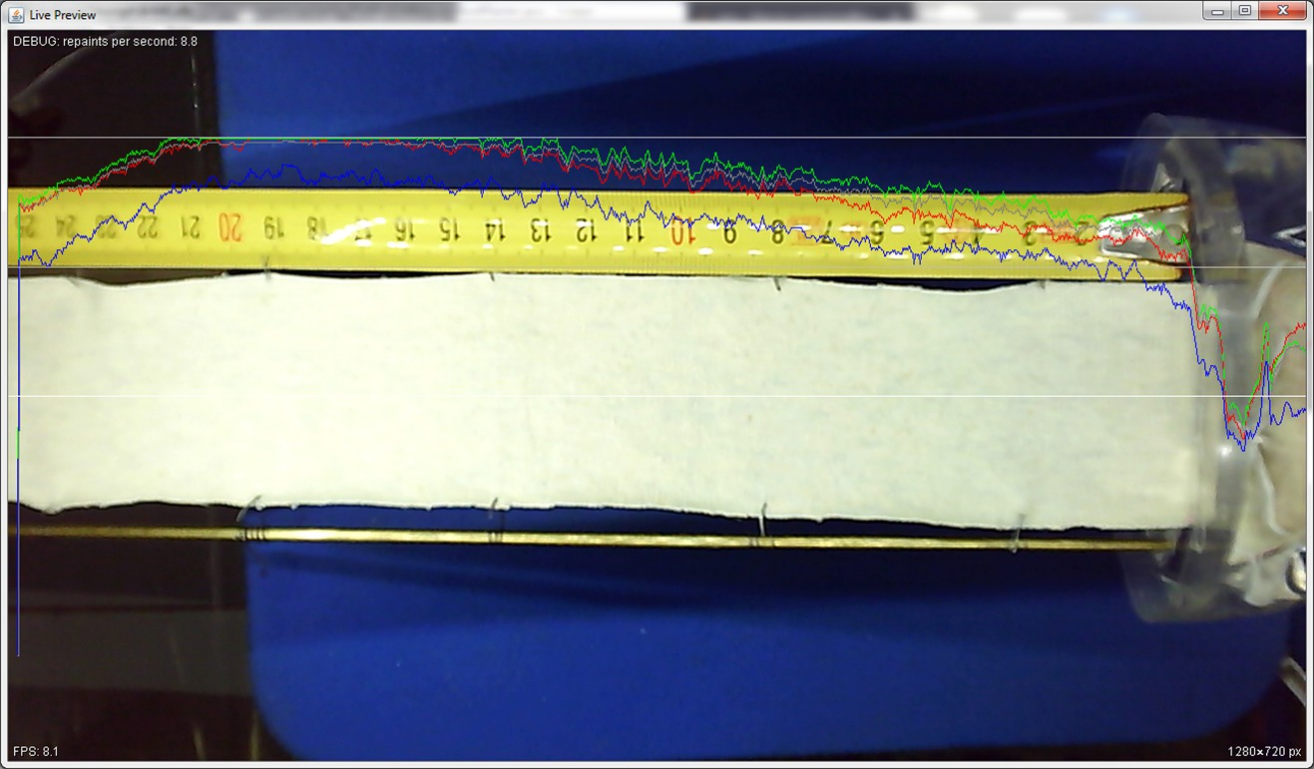
Live preview—example of wrong setting of lighting.

Positioning led strips can be tricky for human eye, and therefore we implemented live preview which helps position the camera and lighting. The graph of intensity of the pixels from the middle of the sample (image), as well as its intensity of red, green and blue color components were added on the current view of the camera. The example of the wrong light setting due to significant variations of mentioned intensities and over-lighting part of the sample (intensity of all components of color are maximal—255) is shown on the Fig 5.

In our experience, in order to synchronize immersing of the sample into water with the image recording, the most appropriate way is to start recording with a frequency of 3 seconds just before sinking, and then to increase in the delay between two images according to the wicking speed.

#### Defining section

**Fig 6:**
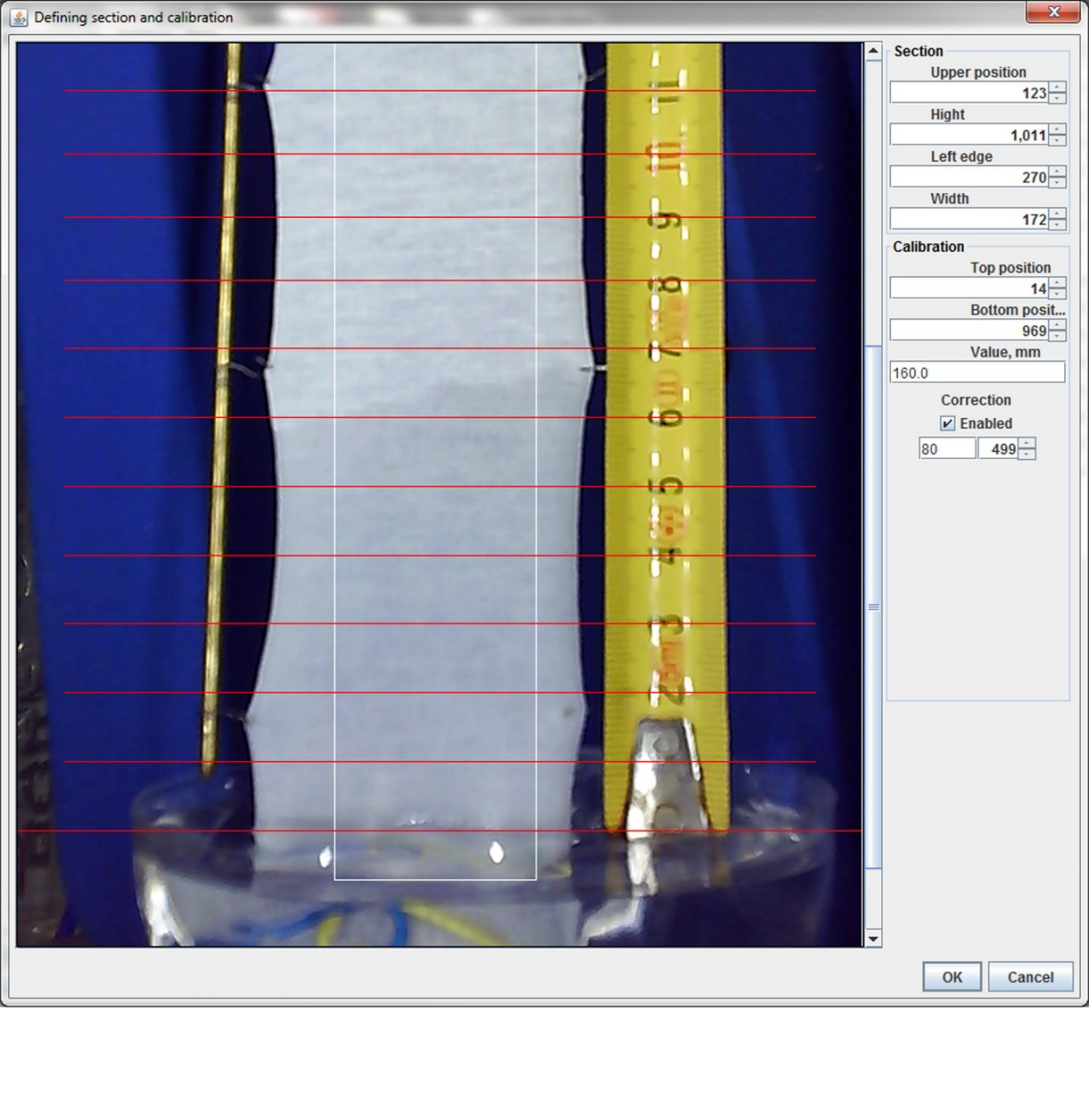
Screenshot of the interface for defining the section and calibration.

In order to define the part of the image of interest, the first step is to select a region of the image which contains a sample and a small part of the liquid in which the sample is immersed. This makes it possible to exclude from the further analysis all parts of the image (background, ruler, construction, etc.) except the sample (Fig 6).

#### Pretreatment–Calibration

In order to obtain the accurate wicking height in mm based on determined height in px, the calibration of the section was performed using a ruler which is placed in the same plane as the sample. Calibration was performed for each sample by defining the position of two values on the ruler. However, we have faced with the effect called barrel-shaped distortion—the magnification of lens system in optical far-axis and that around the optical axis differs from each other causing the points in the image to move back or forth from the optical axis [48]. This effect could be mathematically solved [48], but we got the appropriate accuracy by defining the third point between the bottom point (at the liquid level) and the highest point (Fig 6). Depending on the height of the sample and position of the camera, there was no need for defining the third calibrating point in some cases.

#### Image processing

**Fig 7:**
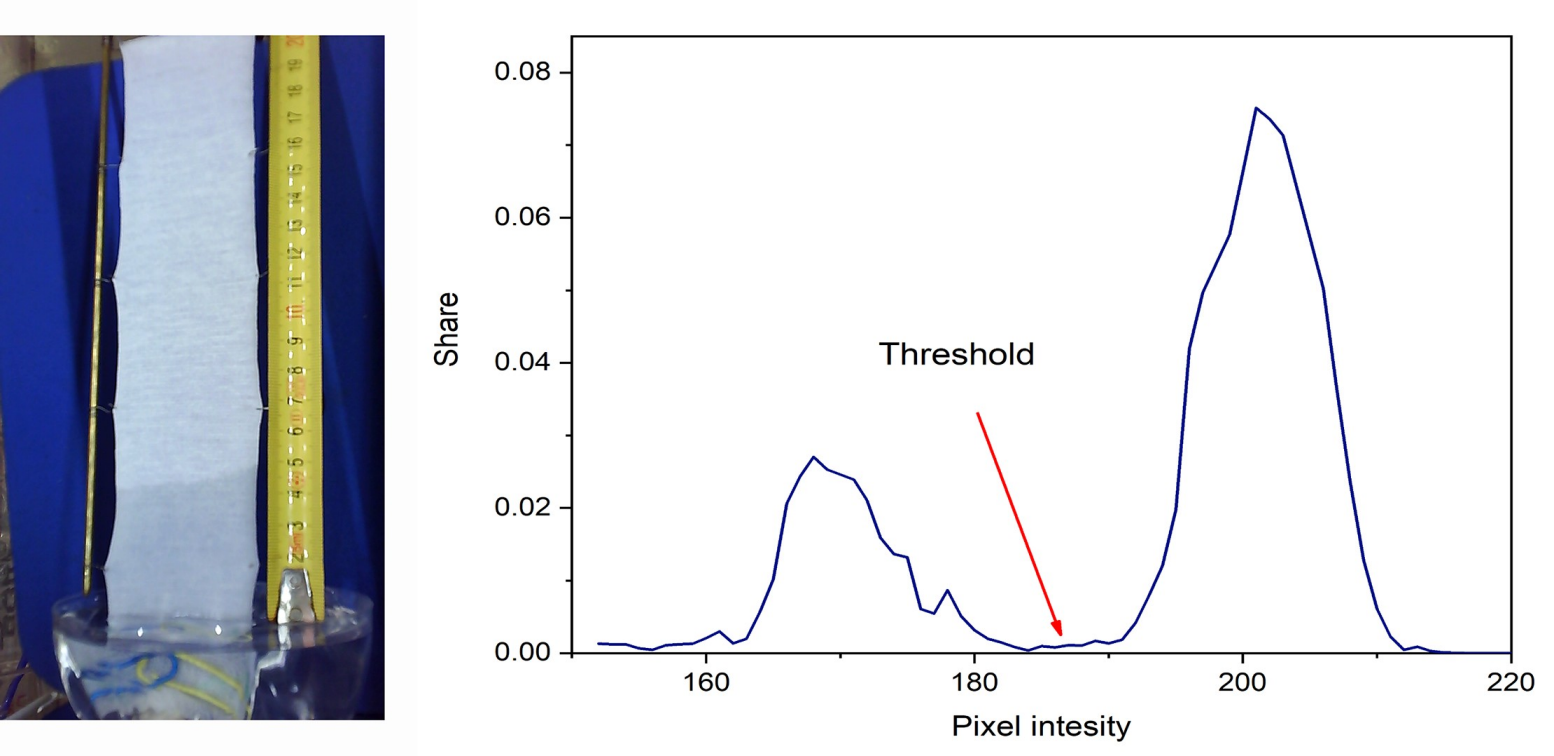
Example of the a) recorded image, and b) histogram of the processed image section during determination of wicking.

The crucial part of this work was to make the difference between the wet and dry part of the sample represented in the section of the image. The selected section of taken image was converted into grayscale, and then the normalized histogram of the gray intensity distribution was calculated. The threshold, which differentiates between the wet and dry part of the sample, is determined based on calculated histogram. The pixels from the wet part of the section are darker and they are presented on the left side of the histogram, while the pixels from the dry part of the sample are lighter and they are located on the right side of the histogram. The threshold is determined by the boundary between the pixel intensity of the wet and dry areas of the sample (the valley of the histogram, Fig 7). Each image section is characterized by its threshold value. In addition, the threshold value slightly increases during the time, and could be greater for 20–40 levels of gray compared to the initial value.

#### Determining the threshold

Determination of the threshold which differentiates the wet from the dry part of the sample is a crucial operation in the process. In order to find out the correct threshold value, we have tried to use the available thresholding methods for developing our own method and to train an artificial neural network for this task. Here is important to point out the time variation of the threshold value (slight increase) resulted from the histogram variation which in turn was caused by changing the ratio of the wet/dry portion of the sample. The results and our conclusions will be presented in this section.

#### The role of ImageJ libraries in determining the threshold

ImageJ is a tool that exists more than 25 years [49], and it is still wildly used by scientist in many areas [50–53]. A lot of published algorithms for determination of threshold were implemented in it. Kapilarko can use all available threshold methods from ImageJ libraries.

**Fig 8:**
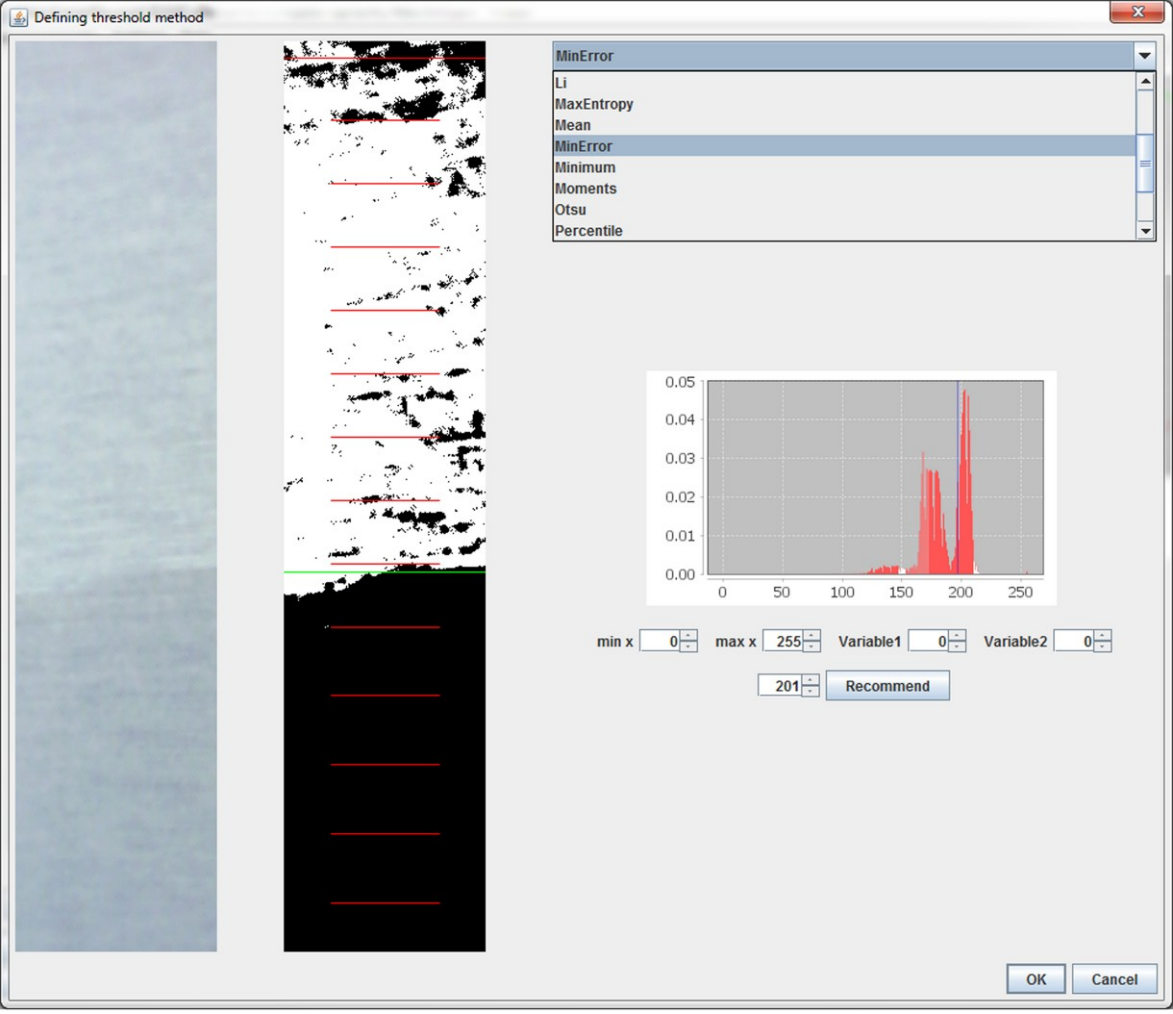
Choosing appropriate threshold method.

Through the user interface, the operator is able to select the implemented ImageJ method which gives the best result for the current image/array of images. Sometimes, one method does not give the correct threshold values for all recorded images during the measurement, and therefore the operator has to use more than one method to cover the whole image series (Fig 8). The operator can choose one or more of the methods from the ImageJ: Default, Huang, Huang2, Intermodes, IsoData, Li, MaxEntropy, Mean, MinError(I), Minimum, Moments, Otsu, Percentile, RenyiEntropy, Shanbhag, Triangle and Yen. The most commonly used method, especially at the beginning of the process of the sample wetting is the Otsu method [54]. This clustering algorithm searches for the threshold which minimizes the intra-class variance, defined as a weighted sum of variances of the two classes [54]. Otsu algorithm is also used by other authors for determination of wicking front [22, 23, 30].

In our experience, the algorithms Huang [55], IsoData [56], Li [57], MaxEntropy, Mean [58], MinError [59], Minimum [60], Moments [61], RenyiEntropy [62] and Triangle [63] give good results, for some time series of images recorded during one experiment.

#### Custom made algorithm for threshold determination

**Fig 9:**
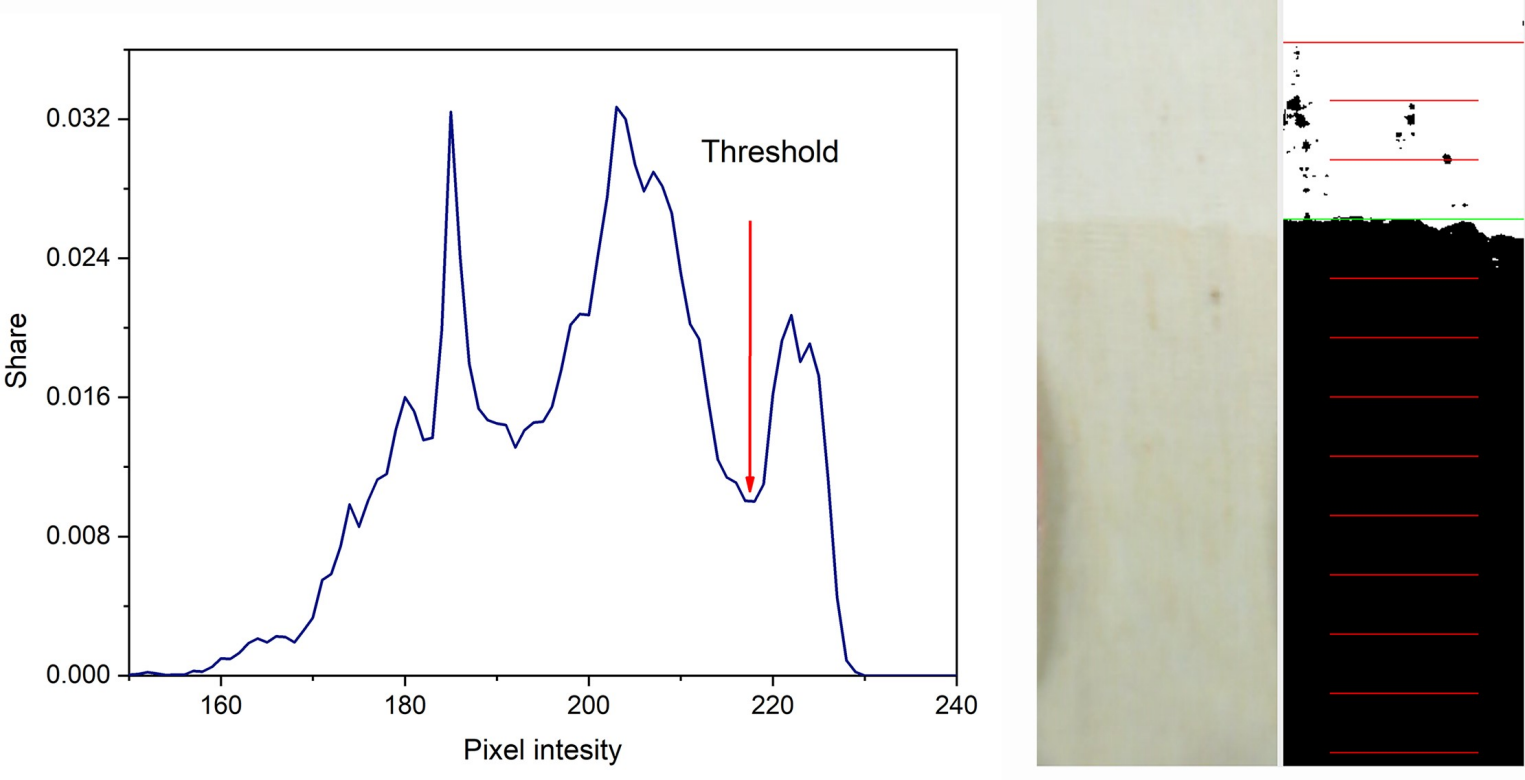
Example of a) “Problematic” histogram, and b) Original and thresholded section.

As the wet and dry areas of the samples are represented with different gray intensity on the gray image, they are separated with the valley that is always noticeable but not always emphasized due to the presence of other valleys (Fig 9A). The most of such situations, we solved by correcting the lighting and defining the section. However, this could not be used when the difference between the wet and the dry portions of the sample is not distinct enough. Since the methods described in the previous chapter could not be always used for the determination of the threshold correctly, we had to define a new algorithm in which four parameters were used for threshold determination. The first two parameters define the scope of the values of gray intensity in which the minimum value of gray intensity (“valley”) is searched. The other two parameters define the minimal depth of the “valley” from the left and from the right side Fig 8. This new method can substitute usage of ImageJ threshold methods and which is more important, can distinguish the wet and the dry areas of the images which are not correctly recognized by ImageJ threshold methods. The disadvantage of this method is that it requires active supervision of the operator during result analysis. The result of the image thresholding with defined threshold is shown in Fig 9B.

#### Reading wicking

**Fig 10:**
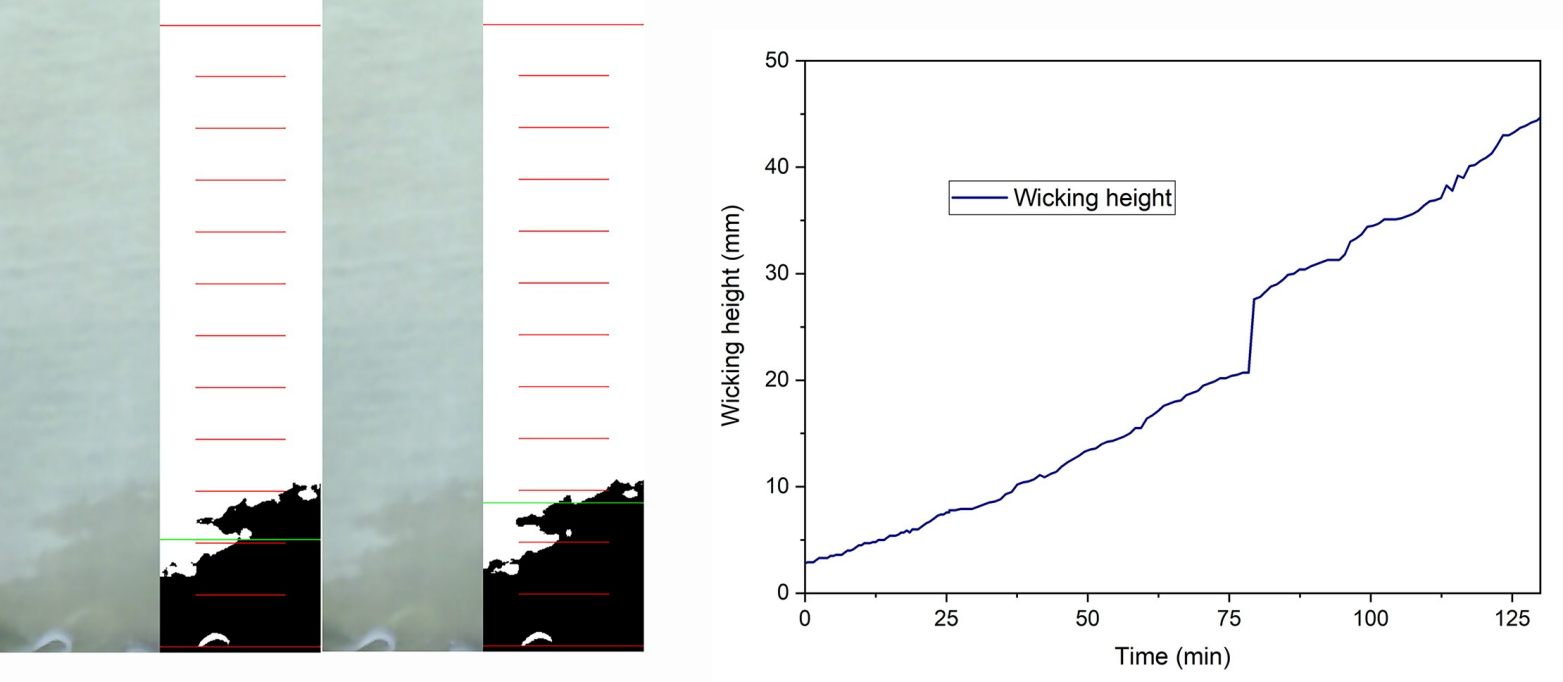
Example of recorded “step” a) Two consecutive images b) Determined graph.

When the calculated threshold is applied, the corresponding wicking height is determined by the level (row) in which at least 50% are dark pixels [48]. The wicking height is determined in pixels and then recalculated in mm based on calibration parameters. If the front line is not a horizontal line, the proportion of the light and dark pixels defines the value of the wicking height. In addition, due to inhomogeneity of the samples, the wicking is very often faster in one of part of the sample (left, right or middle) and the characteristic step can be recognized on the graph. This could be explained by the fact that the number of dark pixels in one row is lower than the threshold (50% of the total number of pixels in one row of the selection) even the maximal wicking height is much greater (Fig 10). When the front is not a horizontal line, the width of the defined section can influence the final result.

### Application of multilayer perception with backpropagation for threshold determination

A histogram was made for each image, based on which the threshold and wicking height were determined. We have investigated the possibility of using trained MLP for process of automatic recognition of wicking to be of additional assistance to the operator. For this purpose we used available tools from the ANN field that are based on the literature data, that shows that result of functions could be obtained by using MLP with one or two hidden layers [64, 65] In this work we have demonstrated that MLP could be trained with histograms on photos of samples and corresponding thresholds, to reliably obtain threshold for determination of the dry and wet part of the samples. Since the determination of thresholds using MLP, in order to identify the wet and dry part of the sample, could be done using basic knowledge and available tools in the field of ANN, we decided to apply them easily, because they fully meet the needs for precision of results and make work even easier for operators. After the sample is processed using procedures described above, the operator should choose representative photos for training by deleting unwanted images, export their histogram data and corresponding threshold value through graphical interface. The next step is defining the parameters for training and testing of MLP– 1. data for training (exported histogram data and corresponding threshold value), 2. testing data (folders with processed samples), 3. defining parameters for network (transfer function, max error, number of hidden layers and numbers of its nodes) and its variations; and starting the parametric analysis. As a result of this analysis, the generated adequate number of files with trained MLPs and the statistical result of testing are obtained. The operator can choose which file with trained MLP will be used in further work.

We decided not to work directly with the images because this would require complicated image processing and it would be much complicated to deal with a calibration due the distortion of the camera lens. Therefore, we decided to train MLP with the histogram data and the threshold value to obtain the threshold value as an output for given histogram data [66]. In order to find optimal architecture of MLP, in this work, we used the parametric analysis with the simplest form of ANN—Multilayer perceptron with Backpropagation with 106 input nodes, one output node and variable numbers of hidden layers and number of nodes in them which will be explained in details.

In order to evaluate the quality of the wicking height prediction using MLP for threshold determination, instead of standard algorithms for image processing described in previous chapter, we used mean squared error (MSE) Eq 1. Mean squared error calculation is based on the expected value of the wicking height (*h_stnd_*—calculated by the steps described in the previous chapter) and the wicking value determined by the threshold calculated by MLP (*h_ANN_*), using the same procedures which are used in “standard” method except that threshold is calculated by MLP.

$$MSE=\frac{1}{n}\sum_{n}\left(h_{ANN}^{2}-h_{stnd}^{2}\right)$$ (1)

It has to be highlighted that MSE is not direct quality assessment of MLP prediction because it also includes the step of calculating wicking height with MLP prediction of threshold. During MLP training the max error is the defined parameter which represents acceptable difference between the threshold values, and it should not be mistaken with MSE value. However, the calculation of MSE is based on the difference between the expected wicking height and the wicking height calculated with the threshold determined by MLP. It is important to clarify this because the values of threshold and wicking height are not linearly dependent. Their correlation is dependent on the histogram shape due the transformation of threshold to the wicking height. In some cases, usually at the beginning of wetting, the difference in the threshold between 5 intensity levels may have no influence on the final result of wicking height. However, in some other cases the difference in the threshold between 3 intensity levels can lead to the difference of 15 mm in wicking height.

### Data preparation for MLP training

Histogram of a grayscale image represents the distribution of the pixel intensity values. For the 8-bit grayscale image there are 256 different possible intensities, and therefore the histogram consists of 256 numbers showing the distribution of pixels amongst those grayscale values [67]. In this work, we used the normalized histogram in order to eliminate the influence of the total number of the image pixels on the histogram values. By this step, we obtained the values in the range of 0–1, which is the range in which MLP can work.

We did not use all the data from the histogram as the input parameters because there is no intensity less than 150 in our grayscale images. In order to reduce the number of input parameters, we used only the normalized histogram values in an intensity range of 150–255. To summarize, in our work with MLP we had 106 input parameters in the range 0–1.

The value of threshold which enabled us to distinguish the wet from dry areas of the sample was chosen for output. Theoretically, this value can vary in the range of 0–255, but as we used the input range of 150–255 the same range was chosen for the output. The output parameter for MLP was calculated according to the Eq 2:

$$Output=\frac{Threshold-{Range}_{min}}{{Range}_{max}-{Range}_{min}}$$ (2)

The output from MLP was the value in the range of 0–1, and the threshold value, which was an integer, was calculated from the output value (function defined above). With the shortening of the used range for the threshold, we reduced the error of transformation of the MLP output into the threshold.

The images used for training were randomly chosen from the available images of the processed sample, bearing in mind that the chosen images should represent the whole range of liquid sorption (beginning, middle, equilibrium). 10–15 images was usually used for training. If the selected images were equally distributed in the array (for example, every fifth image), the slight effect of overlearning was noticed at the end. This effect could be explained by the fact that the frequency of recording is much higher at the beginning than that at the end of the experiment.

### MLP model

A major challenge in the implementation of MLP in the threshold determination is the choice of the appropriate MLP model design involving training algorithms and transfer functions which can yield the smallest error.

There is no general rule for determination of hidden layers number or number of neurons in hidden layers [68–71]. In order to avoid the use of complicated hyperparameter tuning/optimization methods [72–74] for determination of parameters of MLP, and having in mind that we have not expected too complicated structure [66], we have done the parametric analysis. It was done by varying the number of hidden layers (in range 1–3), and by varying the number of nodes with the step of 20 for the first hidden layer, 10 for second hidden layer. We wanted to exclude the situations where the previous layers had less nodes than later layers so they were not omitted in that evaluation.

There have been several types of transfer functions, but in our data set, the values were in the interval between 0 and 1, so we could use only Gaussian or Sigmoid transfer function [75]. Since it was appeared that there was no significant difference between them, we continued with Sigmoid transfer function in our work.

During the parametric analysis, we set the value 0.001 for Max Error, learning rate as 0.02 and limited the maximal number of iterations to 300 000. For every set of the parameters, the calculations were repeated three times with randomly starting coefficients, and there were no significant changes in the final results between them, showing that the design of MLP was appropriate.

The defined number of iterations is not limiting for the tests we have done. The general conclusion is that the addition of more hidden layers or nodes increases learning/processing time, but the accuracy is not thereby much improved. The fact that the number of iterations does not vary too much between repeated trainings, although initial values for the nodes coefficients were chosen randomly, shows that the tested model is appropriate.

**Fig 11:**
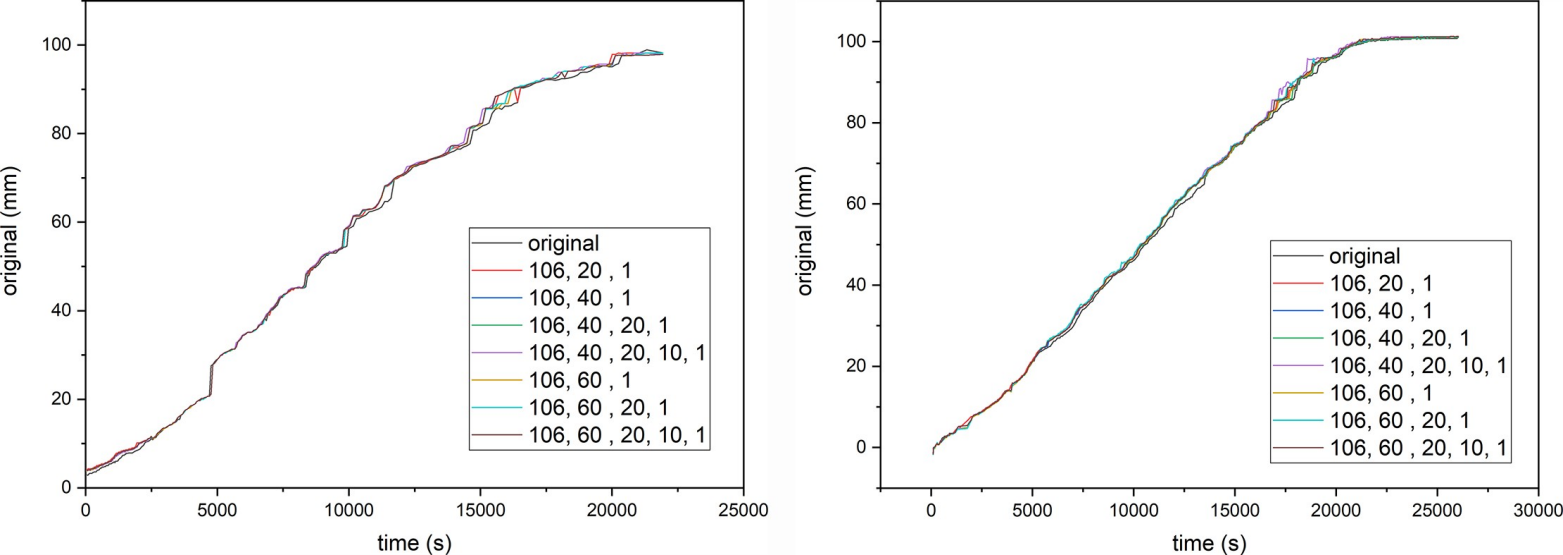
Influence of design on the final result a) Sample 1- bamboo and b) Sample 2- cotton knitted fabrics.

The results of the parametric analysis of a set of images (12 images are chosen for training and all for testing) are presented in Fig 11. For example, the presented results (106, 40, 20, 10, 1) are obtained using MLP for the threshold determination where the structure of MLP is 106 input nodes, 40 nodes in first hidden layer, 20 in second hidden layer, 10 in third hidden layer and one output node.

The results of the parametric analysis are presented in the Table 1.

**Table 1:** Results of parametric analysis (training with data of Sample 1, Max error = 0.001).

Architecture, number of nodes in layers	Repetition	Average training time, s	Number of iterations:	MSE Sample 1	MSE Sample 2
**[106,20,1]**	1	13.1	18,979	3.421	0.708
	2		21,029	2.971	0.509
	3		19,681	3.200	1.670
**[106,40,1]**	1	18.8	15,924	3.524	1.078
	2		14,880	2.970	4.957
	3		13,384	2.877	4.481
**[106,40,20,1]**	1	27.7	18,156	3.763	1.604
	2		14,983	2.849	0.876
	3		19,153	3.253	1.990
**[106,40,20,10,1]**	1	86.2	45,926	3.306	1.056
	2		43,662	3.299	6.110
	3		44,782	2.948	3.676
**[106,60,1]**	1	29.0	13,400	3.390	1.563
	2		14,037	3.265	3.942
	3		14,183	3.248	1.137
**[106,60,20,1]**	1	45.57	15,926	3.117	1.717
	2		16,508	3.060	1.224
	3		17,682	3.241	3.844
**[106,60,20,10,1]**	1	103.0	37,899	2.942	1.579
	2		45,265	3.571	1.312
	3		23,491	3.475	2.324
**[106,60,40,1]**	1	49.8	15,017	3.135	0.715
	2		12,949	2.715	0.640
	3		12,576	2.554	1.799
**[106,60,40,10,1]**	1	95.3	30,623	2.681	2.373
	2		24,360	3.337	0.963
	3		26,893	3.588	1.742

The results presented in Fig 11 and Table 1 were obtained with MLP which was trained with the data for 12 images of the Sample 1 (bamboo knitted fabrics) and tested with all available images of the Sample 1 and the Sample 2 (cotton knitted fabric). It should be mentioned here that during determination of wicking of the Sample 1 only one algorithm (Otsu) was used for the whole range of the experiment, while for the Sample 2 two algorithms (Otsu and MinError) had to be used. Obtained results showed that MLP was not trained to use the only one threshold method, or in other words, it successfully combined more than one thresholding methods.

According to MSE results presented in Table 1 and bearing in mind the fact that tested MPL model was trained with part of data from the Sample 1, it could be concluded that better results were obtained by using the model with data for the Sample 2 than those obtained with data for the Sample 1. In addition, the increased number of hidden layers, as well as number of nodes in them, increases the required number of iteration and training time, but did not have a significant influence on the final results. This can be explained by the fact that the MLP calculates a decimal value for the threshold (in the range 0–1), then the value is transformed in the threshold value (integer in range 150–255) and finally, based on the threshold the wicking height is determined in mm.

The values of wicking height calculated with MLP deviate approximately 1–3.5 mm from the values determined in “standard way”. However, due to the existence of measurement uncertainty of “standard” reading of the wicking height, we can conclude that artificial neural networks can be used as a tool for determination of the wicking height from the sample images.

### Mass recording

During determination of the wicking height, the mass was also recorded. To be precise, the recorded mass was the mass of the fluid in the vessel which decreased with time. The loss of weight was obtained due to the evaporation of water in the vessel, adsorbed water mass by a sample (textile structure) and evaporation of water from the sample [21]. Mass was recorded in 2 second intervals and simultaneously shown on the graph on the main screen.

**Fig 12:**
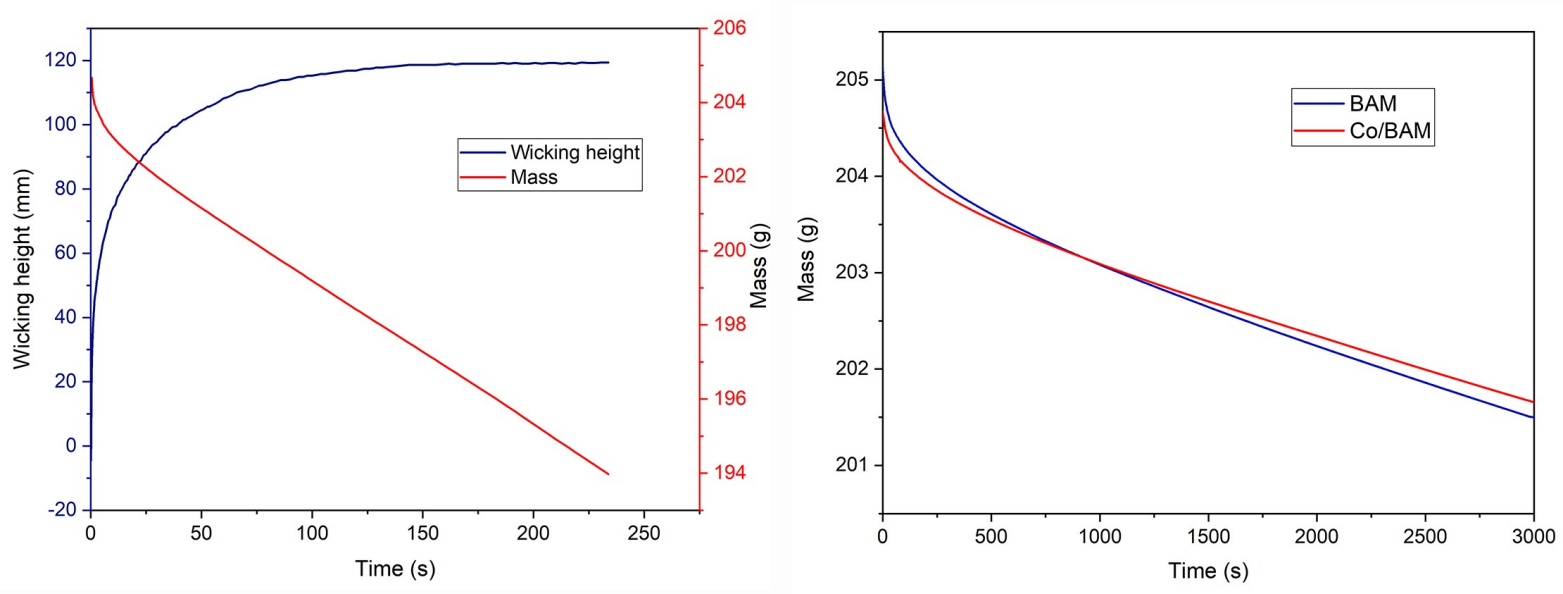
Example of a) determined wicking height of the hemp knitted fabric (5 cm with) and mass recorded b) comparison of mass decrease in vessel for untreated and treated fabric.

Fig 12A) shows an example of a graph with recorded mass and determined wicking height. Fig 12B) gives the comparison of recorded liquid mass decrease in vessel for the untreated and treated fabrics–there is a slightly faster mass decrease with treated fabric due to its higher wicking height. It is important to highlight that Kapilarko is able to provide data for calculating the speed of water evaporation from the vessel without a sample, which is necessary for further mathematical/numerical determination of the coefficients of evaporation from textile structures and the coefficient of liquid adsorption. These functions have not been implemented yet, but this is our next goal. These coefficients depend on the ambient conditions, and therefore the monitoring of temperature and humidity during the measurement will be essential.

### Demonstration of device’s utility

**Fig 13:**
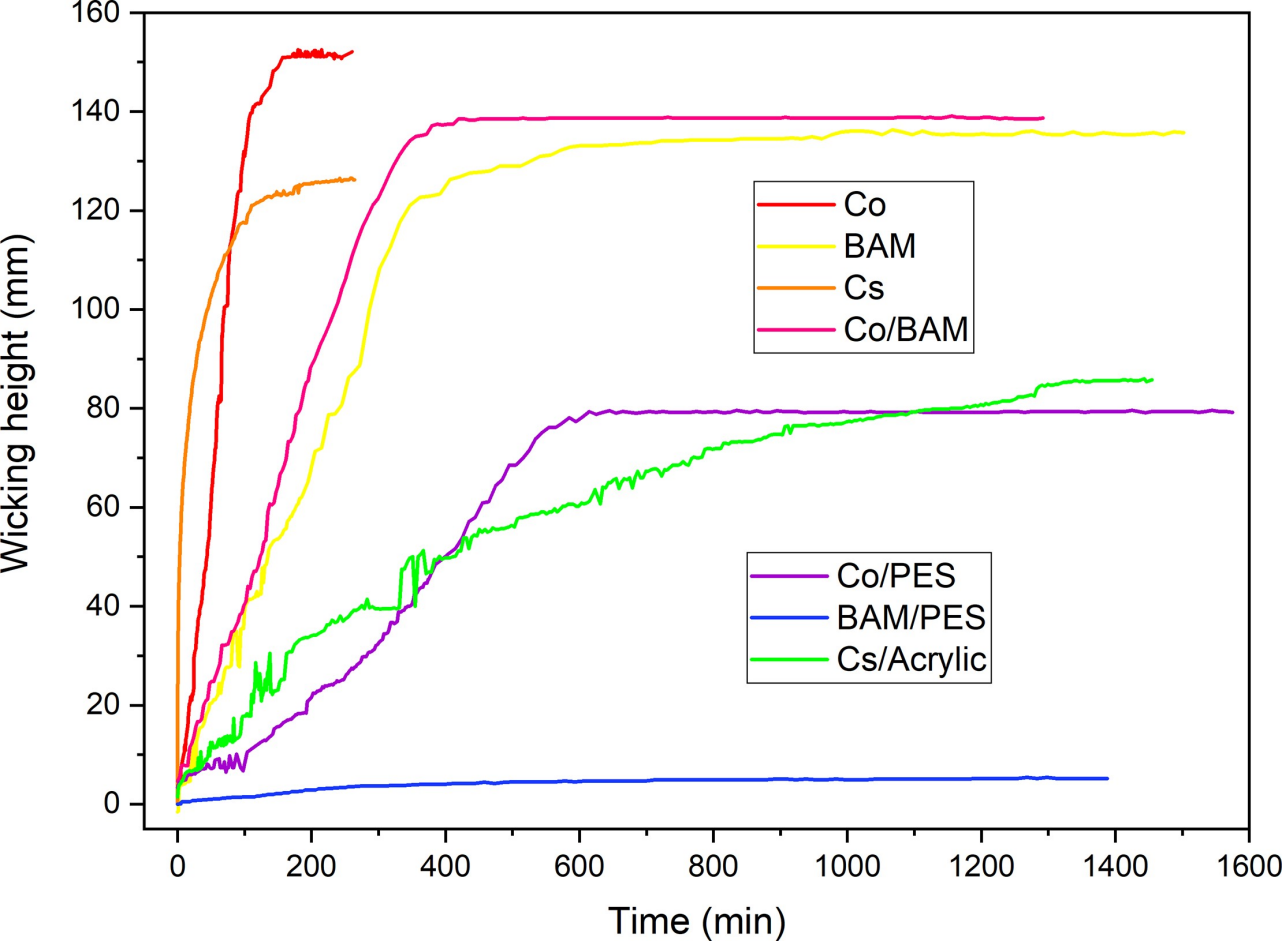
Example of experimental determination of the wicking height of different knitted fabrics (Co-Cotton, BAM-Bamboos, Cs-Hemp), and blended composition (Co/BAM, Co/PES-polyester, BAM/PES and Cs/Acrylic).

Fig 13 shows several examples of monitoring the capillary flow of water and determined wicking height of different knitted fabrics. In our next paper, the influence of composition, properties and processing of knitted fabrics on their wicking ability will be discussed in detail. Now we want to show that the device presented in this paper can be successfully applied for determination of the wicking ability of textile fabrics, either those for which the wicking height is reached relatively quickly (Fig 13, cotton, red line), or those for which much time is taken to reach max wicking height (Fig 13, hemp/acrylic, green line). The graph shows not only the curves with a "good" reading of the height of the fluid progression (e.g. blue and pink lines), but also the “jump curves" (green line), the reason for which is previously explained. For each tested sample, there is also a set of photographs which can be used to make the video of the capillary action. An example of the video of the wicking progression for the cotton/bamboo knitted fabric is given in S1 Video.

## Conclusion

Connection of the available laboratory equipment (computer, balance, web camera), with designed open-source software “Kapilarko”, ImageJ program for image processing, and new algorithm, all together, resulted in the development of an automated method and construction of the device for determination of wicking ability of textile structures which has a number of advantages. Some of them are automated measurement and minimization of the subjective errors of the operator; extremely fast as well as long-term measurements; digital recording of the results; consistency of the experimental conditions; possibility of using water for measurements instead of colors; low cost of the experimental settings.

In addition, we have successfully trained artificial neural networks for determination of threshold which is used for recognizing the wet and dry areas of samples with the aim of automatic recognizing of the wicking height. Obtained results have shown that the procedure of recognition of sample’s wet area is in very good agreement with the experimental data.

Taking into consideration the importance and frequent need for determination of the wicking ability of textile materials, all mentioned advantages of presented device as well as the fact about the availability of the open-source scientific tools, other researchers can redesigned and adapt the wicking device to their needs, there is no doubt that presented device with "open-source" approach, will be useful in many aspects for improving experiments for the determination of the wicking properties of textile structures. Furthermore, we believe that this device can be also used for determination of the wicking ability of other porous polymer or non-polymer structures (foams, paper and composites).

## Supporting information

S1 Fig Photo of the experimental set. Photo of the experimental set that was used.
(DOCX)

S1 File Graphical User Interface (GUI). Screenshots of the Java GUI that represent functionality of “Kapilarko”.
(ZIP)

S2 File Exported data. Example of exporting data to excel file.
(XLSX)

S1 Video Generated video. Generated video from recorded images.
(MP4)
