# Supplementary figures and images for: Development of the automated software and device for determination of wicking in textiles using open-source tools
Source: PLoS One. 2020 Nov 16;15(11):e0241665. doi: 10.1371/journal.pone.0241665 (PMC7668598; doi:10.1371/journal.pone.0241665)

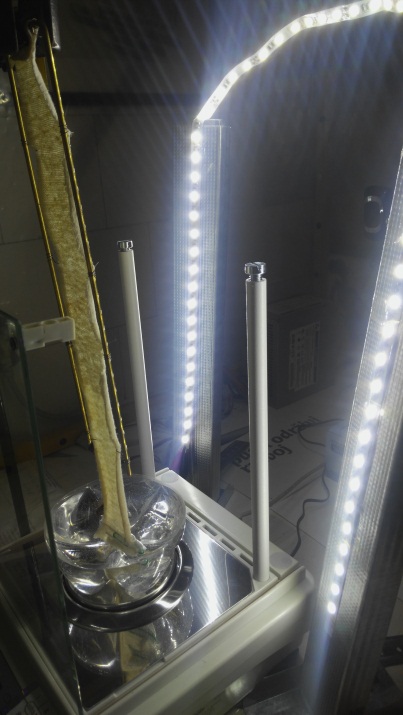


**S1 Fig.** Photo of experimental set that was used for wicking determination

Supplement: S1 Fig — Photo of the experimental set that was used. (DOCX) [file pone.0241665.s001.docx]

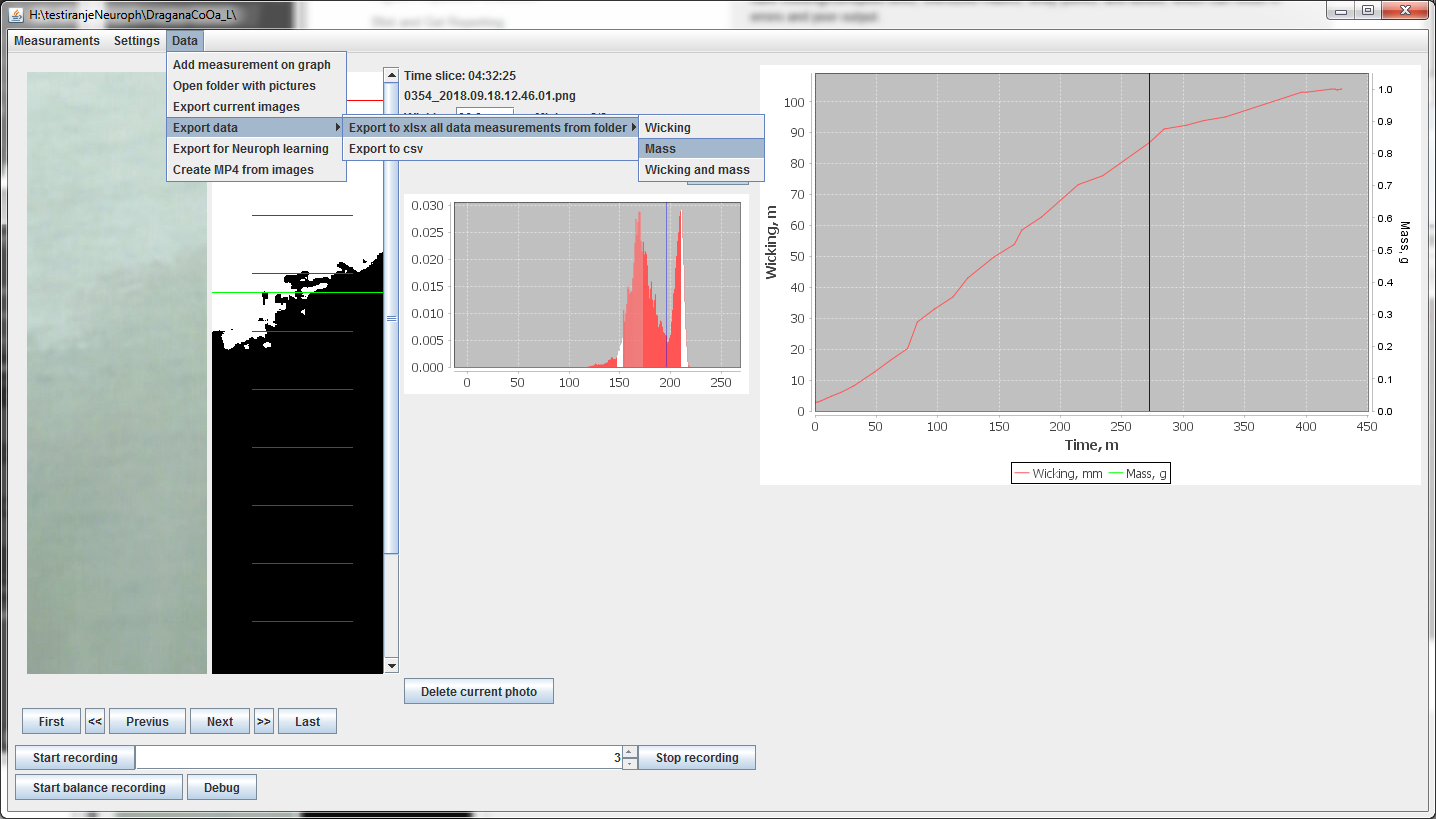

Supplement: S1 File — Screenshots of the Java GUI that represent functionality of “Kapilarko”. (ZIP) [file pone.0241665.s002.zip › Fig D The menu of main 3.png]

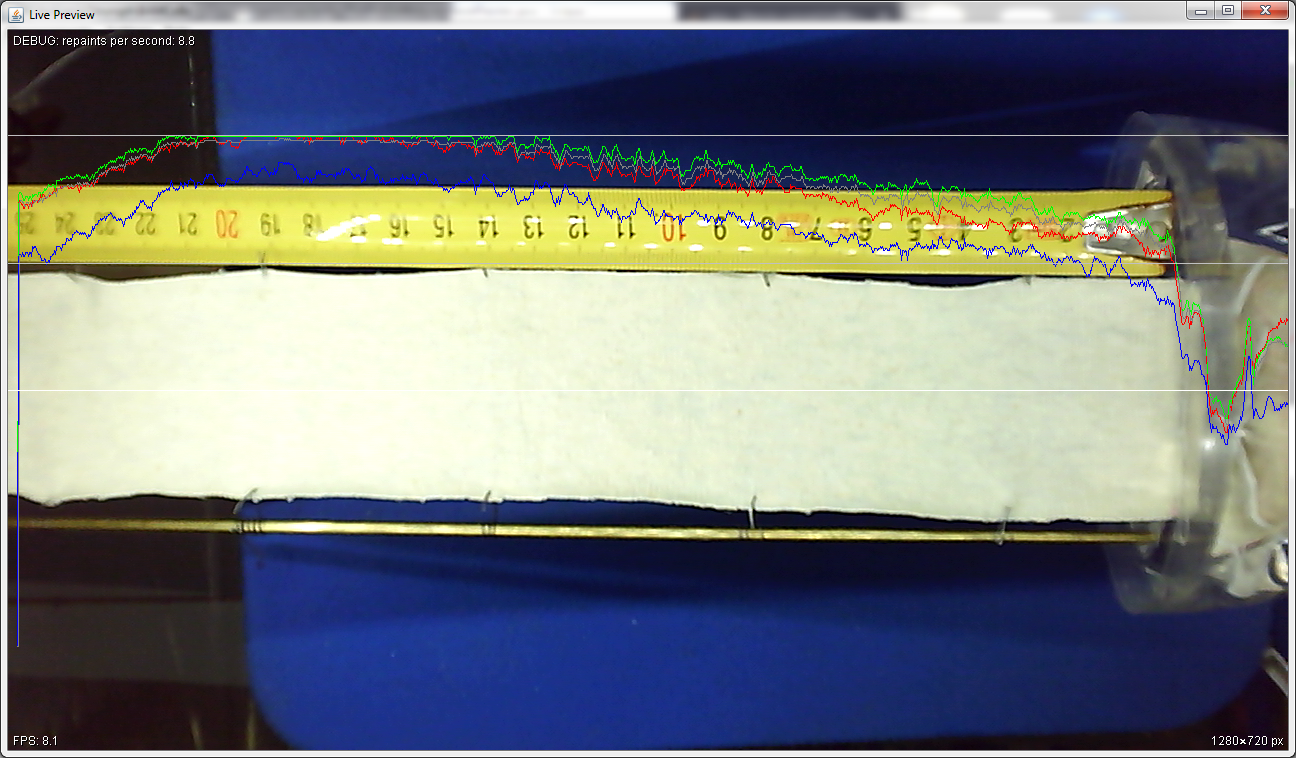

Supplement: S1 File — Screenshots of the Java GUI that represent functionality of “Kapilarko”. (ZIP) [file pone.0241665.s002.zip › Fig E The winodow with live preview.png]

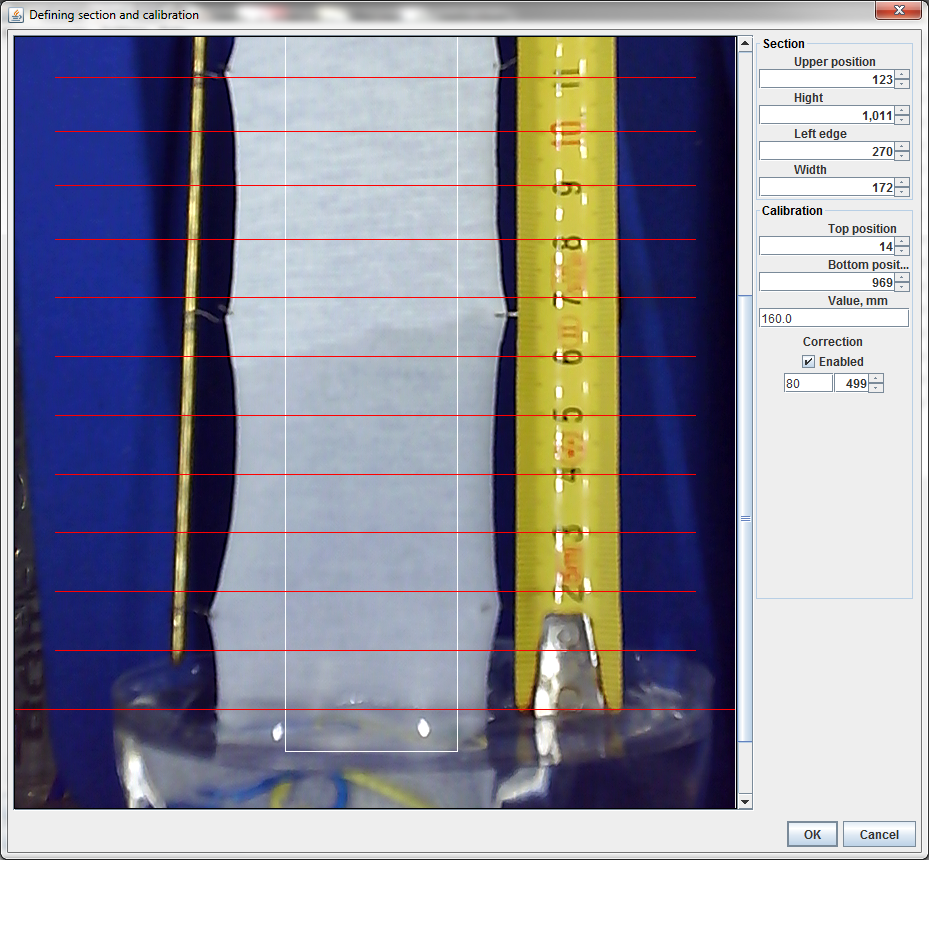

Supplement: S1 File — Screenshots of the Java GUI that represent functionality of “Kapilarko”. (ZIP) [file pone.0241665.s002.zip › Fig F The interface for defining section and calibration.png]

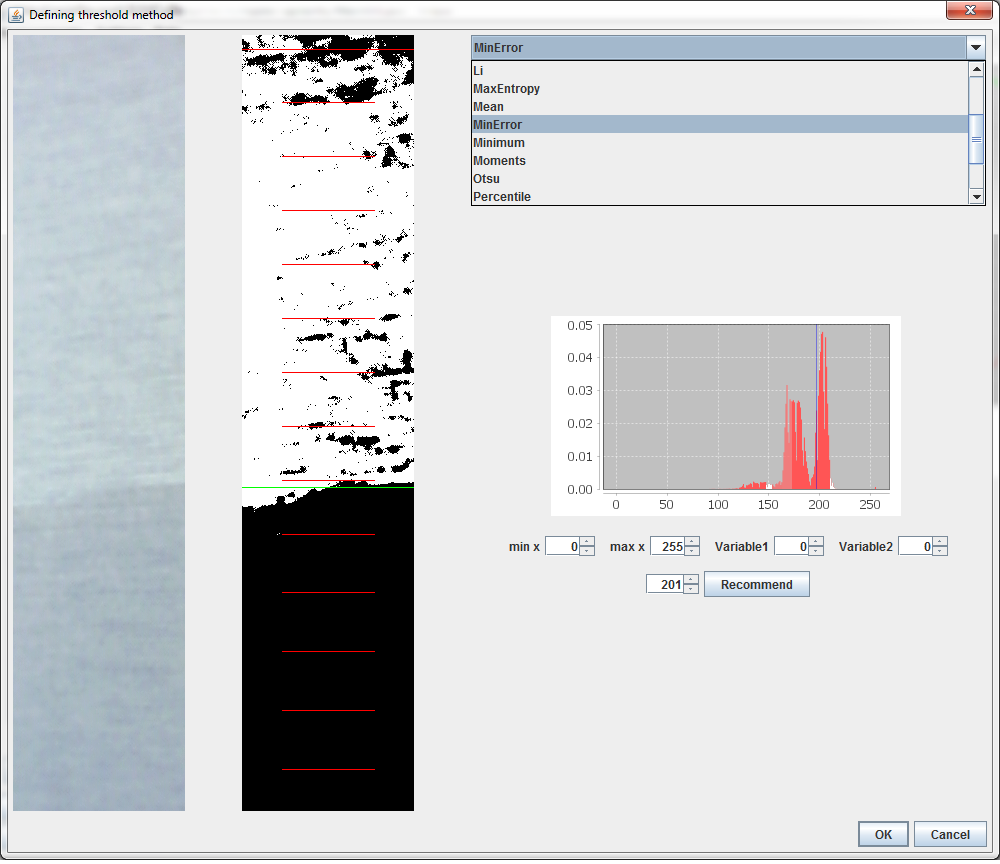

Supplement: S1 File — Screenshots of the Java GUI that represent functionality of “Kapilarko”. (ZIP) [file pone.0241665.s002.zip › Fig G The window for defining threshold method.png]

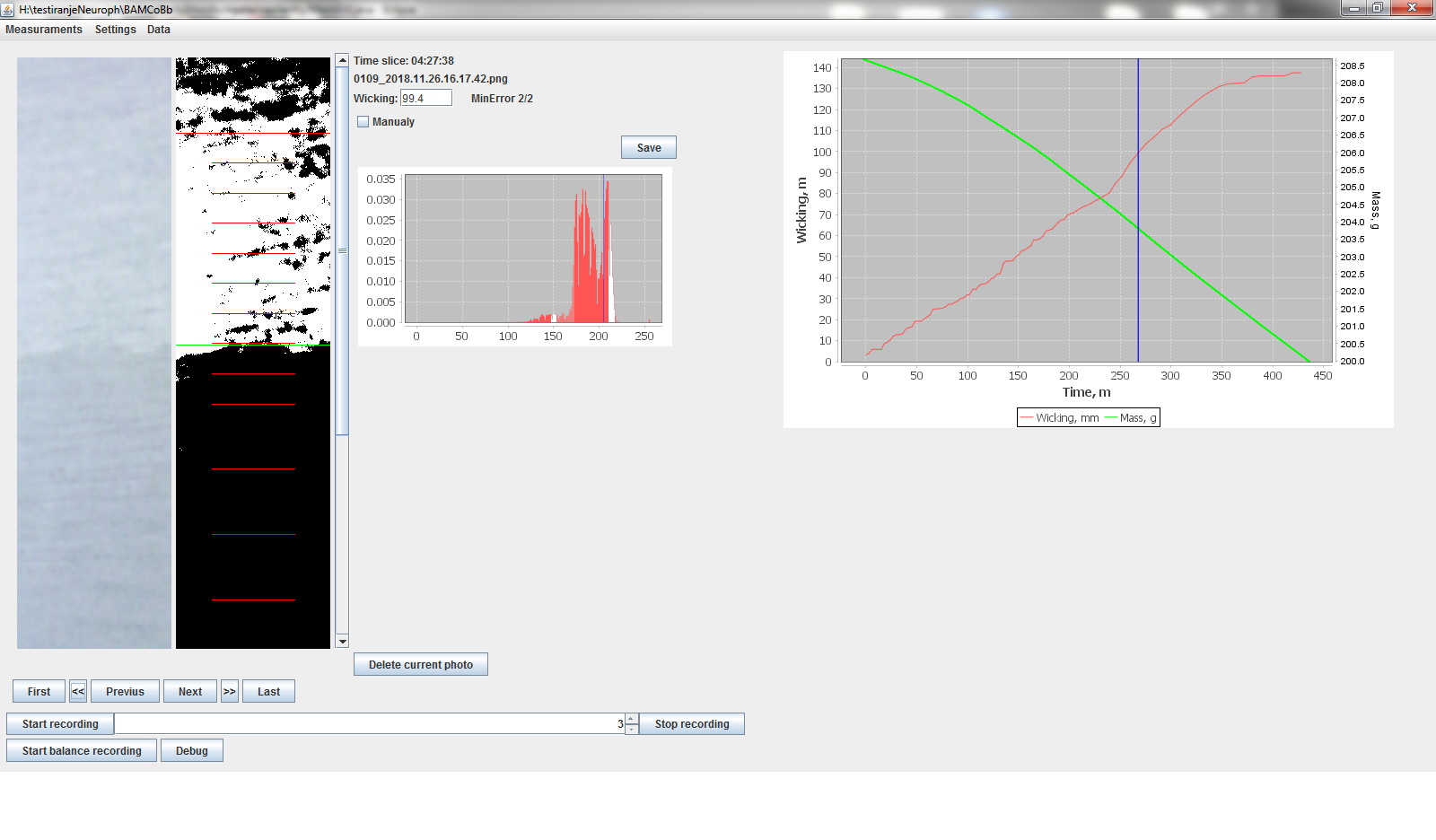

Supplement: S1 File — Screenshots of the Java GUI that represent functionality of “Kapilarko”. (ZIP) [file pone.0241665.s002.zip › Fig A The main window during work.png]

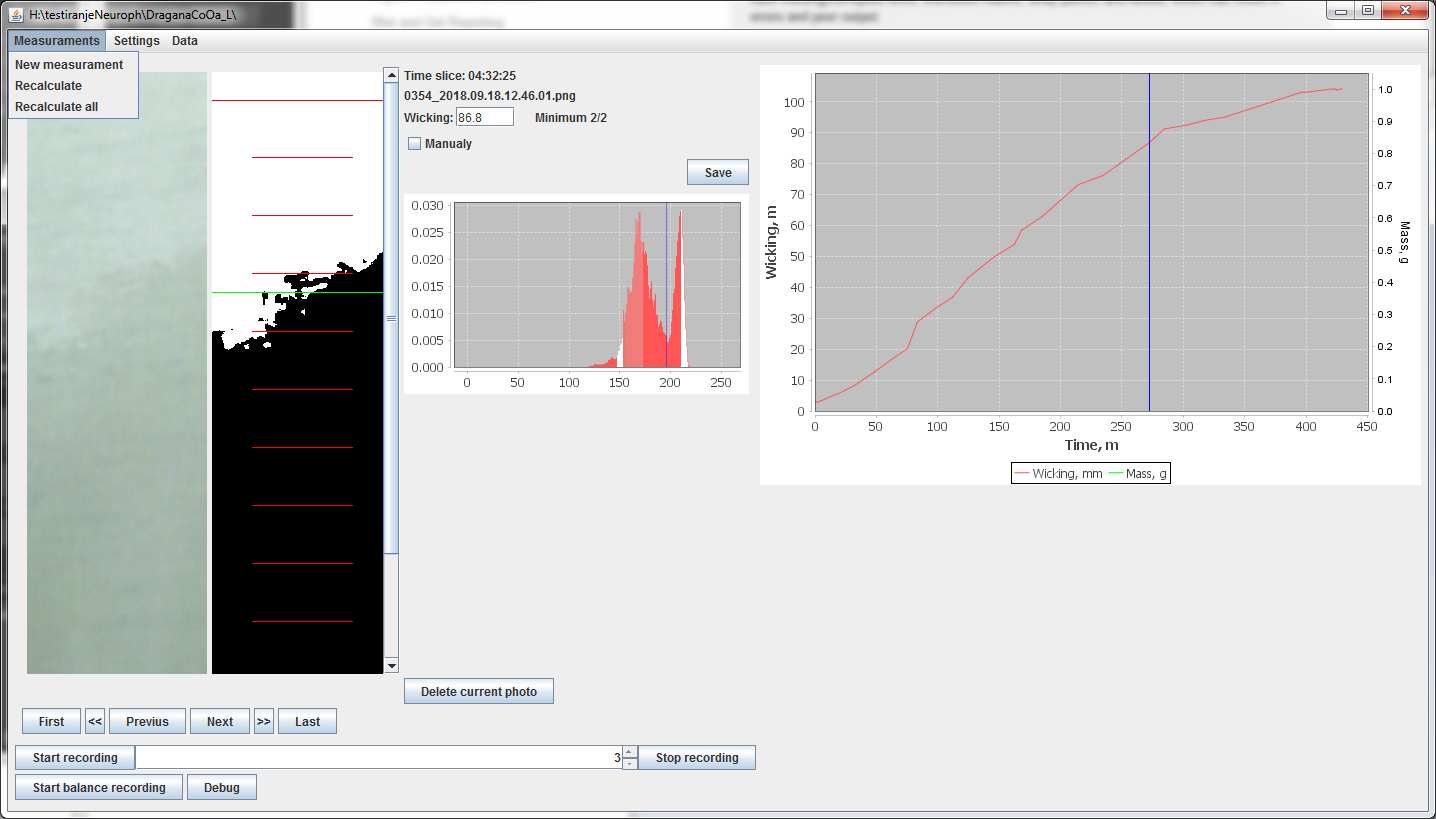

Supplement: S1 File — Screenshots of the Java GUI that represent functionality of “Kapilarko”. (ZIP) [file pone.0241665.s002.zip › Fig B The menu of main 1.png]

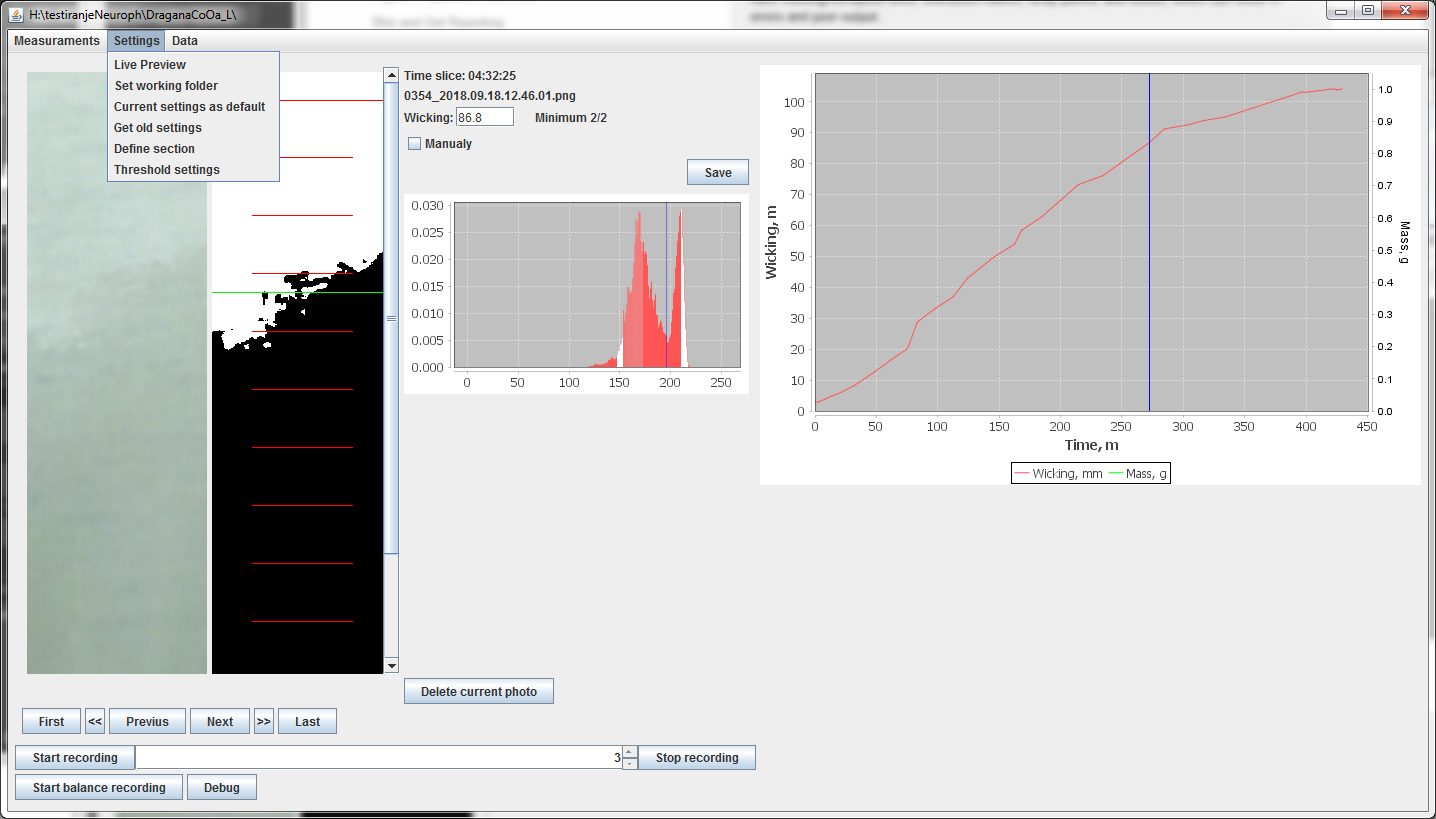

Supplement: S1 File — Screenshots of the Java GUI that represent functionality of “Kapilarko”. (ZIP) [file pone.0241665.s002.zip › Fig C The menu of main 2.png]
